# IL-10RA governor the expression of IDO in the instruction of lymphocyte immunity

**DOI:** 10.1038/s41416-024-02893-3

**Published:** 2024-11-26

**Authors:** Tzong-Shyuan Tai, Duen-Wei Hsu, Yu-Shao Yang, Ching-Yen Tsai, Jai-Wen Shi, Chien-Hui Wu, Shu-Ching Hsu

**Affiliations:** 1 Department of Medical Research and Development, Chang Gung Memorial Hospital, Taoyuan, Taiwan; 2 Department of Biotechnology, National Kaohsiung Normal University, Kaohsiung City, Taiwan; 3 National Institute of Infectious Diseases and Vaccinology, National Health Research Institutes, Miaoli, Taiwan; 4 Institute of Bioinformatics and Structural Biology, National Tsing Hua University, Hsinchu, Taiwan; 5 Transgenic Core Facility, Institute of Molecular Biology, Academia Sinica, Taipei, Taiwan; 6 Department of Surgery, National Taiwan University Hospital, Taipei, Taiwan; 7 Graduate Institute of Medicine, College of Medicine, Kaohsiung Medical University, Kaohsiung City, Taiwan; 8 Graduate Institute of Biomedical Science, Immunology Research and Development Center, China Medical University, Taichung City, Taiwan; 9 Department of Biomedical Sciences and Engineering, Tzu Chi University, Hualien, Taiwan; 10 Doctoral Program in Tissue Engineering and Regenerative Medicine, National Chung Hsing University, Taichung City, Taiwan

**Keywords:** Cancer microenvironment, Target identification

## Abstract

**Background:**

Indoleamine 2,3-dioxygenase (IDO) impairs anti-pathogen and anti-tumour immunity. Mesenchymal stem cells (MSCs) modulate immunity via IDO but also suppress IFN-γ. While MSC IDO induction by IFN-γ is established, other drivers in this immunosuppressive setting remain unknown.

**Methods:**

Human bone marrow mesenchymal stem cells (MSCs) with IDO or IL-10RA knockdown were co-cultured with healthy donor T cells to assess immunosuppression. PDAC organoid anticancer activity was also tested in these co-cultures.

**Results:**

Co-culturing MSCs with T cells in an IL-10RA-enriched environment enhances IDO expression, resulting in T cell suppression. Moreover, IL-10RA-positive MSCs collected from co-cultures with IL-10 supplementation show increased IDO expression. Conversely, MSCs with IL-10RA knockdown exhibit a significant reduction in IDO RNA and protein expression, as well as STAT3 phosphorylation status, which is a known upstream signalling pathway in IDO gene regulation, in T cell co-cultures. Down-regulation of IL-10RA also inhibits IDO activity in MSCs, resulting in reduced T cell suppression, and enabling the co-cultured T cells to kill PDAC organoids.

**Conclusion:**

Our research reveals IL-10RA as a pharmacological target in stromal cells for enhancing T cell-mediated PDAC eradication by downregulating IDO via blocked IL-10/IL-10RA signalling in MSCs. This advances IL-10RA interference in the tumour microenvironment (TME) to restore T cell cytotoxicity against cancers.

## Introduction

L-tryptophan, an essential amino acid, undergoes conversion to kynurenine primarily through the initial and rate-limiting activity of indoleamine 2,3-dioxygenase (IDO), a crucial player in antimicrobial and antitumor responses with demonstrated immunomodulatory activity [1, 2]. IDO has been proven as a critical factor in the immunosuppressive mechanism of human or non-human primate mesenchymal stem cells (MSCs) (stromal cells), dendritic cells (DCs), and tumour cells [3]. The elevated expression of IDO across various cell types is postulated to induce immune system suppression by depleting local tryptophan storage and accumulating downstream kynurenine metabolites, thereby impeding T cell proliferation and suppressing T cell function [4]. Within the tumour microenvironment, both IDO-expressing DCs and tumour cells contribute to immune evasion, thus facilitating tumour development [5–7]. Consequently, IDO has emerged as a promising target for immunomodulation. Ongoing advancements in the field involve the development of diverse therapeutic interventions targeting IDO, frequently in combination with immune checkpoint inhibitors (ICI), for various cancers [8, 9]. However, the setback observed in the clinical trial of an IDO inhibitor underscores the imperative for a more comprehensive understanding of IDO’s role in immuno-oncology [10].

Derived from the early mesoderm, MSCs constitute a heterogeneous group of cells with notable capabilities for self-renewal and multidirectional differentiation [11, 12]. MSCs exhibit a high proliferative capacity along with potent immunomodulatory capabilities [13]. Consequently, the immunomodulatory attributes of MSCs provide a therapeutic avenue for T cell-mediated diseases in regenerative medicine, including multiple sclerosis (MS), graft-versus-host disease (GvHD), and the prevention of organ transplantation rejection [14, 15]. MSCs have also been observed to impede the maturation of antigen-presenting cells (APCs) and regulate T cell responses via the IL-10R-mediated interaction with IL-10 [16]. IL-10 engineered MSCs have been shown to enhance suppression or exhibit anti-inflammatory activity in models of GvHD or spinal cord injury [17, 18]. In the immune microenvironment, IDO and IL-10 both demonstrate suppressive responses and are frequently present simultaneously [19, 20]. In an allograft transplantation model, MSCs demonstrated immunosuppressive effects through elevated expression of IDO and IL-10 in the graft microenvironment [21].

Originally identified as a cytokine derived from Th2 cells, interleukin-10 (IL-10) operates by inhibiting the activation and cytokine production of Th1 cells [22]. Subsequently, the cellular origin of IL-10 has significantly expanded to encompass various cell types, notably B cells and T cells. In contrast, IL-10 receptor complex, essential for mediating IL-10’s biological effects, is characterised as a hetero-tetramer comprising IL-10RA and IL-10RB subunits. While IL-10RA is selectively expressed in hematopoietic lineages (such as T cells, B cells, NK cells, mast cells, and dendritic cells), the IL-10RB subunit is ubiquitously expressed throughout the body [23, 24]. Upon IL-10 engaging, phosphorylation of tyrosine residues on IL-10RA serves as pivotal docking sites, predominantly orchestrating the recruitment and activation of the transcription factor STAT3 [25]. The IL-10/IL-10 axis plays a crucial protective role in autoimmune disorders, as illustrated in conditions like MS and inflammatory bowel disease (IBD). This manifestation is evident in both IL-10 knockout mice and patients with mutations in IL-10 and its receptors [26–28]. These findings highlight the fundamental importance of IL-10 signalling in maintaining immune homoeostasis. However, the regulatory interplay between the IL-10 pathway and IDO remains relatively unexplored.

The existence of interferon-stimulated response element (ISRE) on the *IDO* gene renders Interferon-γ (IFN-γ) a potent inducer for IDO expression [29]. MSCs have been demonstrated the immunoregulatory capability for inhibition of T cell proliferation with IFN-γ treatment [30]. Nevertheless, considering the heterogeneity of stromal cells within the immunosuppressive microenvironment, the existence of IFN-γ-independent pathways could potentially contribute to an effective immunomodulatory response. Modulating MSCs activity has shown promising benefits in specific tumour models [31, 32], suggesting that a more profound understanding of the immunosuppressive activity of MSCs has the potential to pave the way for enhanced cancer therapies. In this study, we identified the regulation of IDO responses in MSCs through IL-10/IL-10R/STAT3 axis. We observed an up-regulation of IL-10RA expression on MSCs upon co-culture with activated T cells. Engagement of the IL-10/IL-10RA axis propagated IDO expression in MSCs, altering the physiological functions of T lymphocytes. Our findings suggest that targeting IL10RA expression could offer a novel approach for cancer therapy by reducing IDO levels in the tumour microenvironment (TME), thereby facilitating the reversal of T cells-mediated killing of cancer cells.

## Material and methods

### Human BM-MSCs culture

Human bone marrow-derived mesenchymal stem cells (MSCs) were purchased from Lonza Walkersville Inc. (PT-2501, Lonza Walkersville Inc.). The identity of MSCs was confirmed through the examination of surface marker expression using flow cytometry (Supplementary Fig. 1). Cells were cultured in Iscove’s modified Dulbecco’s medium (IMDM, Hyclone) supplemented with 10% fetal bovine serum (FBS, Thermo Fisher Scientific), 10 ng/mL basic fibroblast growth factor (bFGF, Peprotech), 100 U/mL penicillin, 100 μg/mL streptomycin, and 2 mM L-glutamine (Invitrogen) and maintained at 37 °C incubators with 5% humidified CO_2_. In this study, only the MSCs between passages 6 to 9 were used. Furthermore, the use of MSCs was granted approval by the Research Ethics Committee, National Health Research Institutes (EC1111003-W).

### Purification of human CD3^+^ T cells

Human T cells were obtained from peripheral blood from healthy donors. PBMCs were freshly purified by Ficoll-Paque PLUS density gradient media (Cytiva). Untouched CD3^+^ T cells were then purified from the isolated PBMCs using a human Pan T cell isolation kit (Miltenyi Biotec, Cat# 130-096-535). The purity of the human CD3^+^ T cells was greater than 95%. The use of PBMCs was also approved by the Research Ethics Committee, National Health Research Institutes (EC1111215-W).

### Co-culturing of MSCs and T lymphocytes

T cells (1 × 10^5^) were treated with or without 5 μg/mL of phytohemagglutinin (PHA, Sigma-Aldrich) in the absence or presence of 1 × 10^4^ or 2 × 10^4^ MSCs for 3 days. To separate MSCs from T cells, the suspension T cells were carefully collected following co-culture. The plate was subsequently washed three times with PBS, and the attached MSCs were then harvested. Cell purity was evaluated using flow cytometry, revealing a purity of greater than 90% (Supplementary Fig. 2A).

### Thymidine incorporating assay

For the [^3^H]-thymidine incorporating assay, 1 μCi of [^3^H]-thymidine was added after 72 h of incubation. Cells were harvested using a Filtermate 96-well harvester followed by an additional 16 hours of incubation. The radioactivity (CPM) was measured to quantify cell proliferation using a Packard microplate scintillation and luminescence counter (Perkin-Elmer-Packard, USA).

### MSCs viability and suppressive activity with STAT3 inhibitor 4 m

MSCs were seeded in 96 well plates (5000 cells/well) with varying doses (0.1, 0.5, 1, and 10 μM) of STAT3 inhibitor 4 m (Cayman, Cat#37352) for 3 days. Cell viability after treatments was further measured using WST-1 assay kit (Sigma-Aldrich, Cat# 11644807001) according to the manufacturer’s instruction. The absorbance at 450 nm was monitored using SpectraMax® M2/M2e microplate reader (Molecular Devices, Inc.) with the reference wavelength set at 630 nm. Relative cell viability for each group was expressed as a percentage compared to the vehicle control (Supplementary Fig. 3). For the test of STAT3 inhibitor, MSCs were co-cultured with T cells as indicated. On day 2 of co-culture, T cells were washed out, the medium was refreshed, and 1 ng/mL of IL-10 and 0.1 μM of STAT3 inhibitor 4 m or vehicle were added to the MSCs and culture for an additional day. After 24 hours, the conditioned medium was collected and subjected to the kynurenine assay to evaluate IDO activity.

### T cells proliferation and activation assay

For proliferation assay, purified T cells were labelled with CellTrace™ CFSE Cell Proliferation Kit (Invitrogen) and co-cultured with or without MSCs in the presence or absence of PHA for 3 days. Cells were then stained with an anti-CD3-APC antibody (BioLegend, Cat#317318). The percentage of CD3^+^ proliferating T cells, identified as CFSE-attenuated cells, was collected using a Cytek Aurora flow cytometer (Cytek) and analysed by FlowJo software (10.8.0). For investigation of the T cell activation marker, co-cultured T cells were stained with anti-CD3-FITC (BD bioscience, Cat#566783) and anti-CD69-AF647 (Invitrogen, Cat#MA5-18150) before FACS analysis.

### IDO activity assay

The biological activity of IDO can be determined by measuring the level of kynurenine in culture supernatants modified from previously described [33]. Briefly, culture supernatants, or standard samples defined kynurenine concentration (0–160 μM), were mixed with 30% trichloroacetic acid (Sigma-Aldrich) and centrifuged at 2000 × *g* for 10 min. Mixed 75 μL of the suspension with an equal amount of Ehrlich reagent. Absorbance at 492 nm was measured to determine kynurenine concentration based on a standard curve.

### Enzyme-linked immunosorbent assay (ELISA)

Supernatants from the co-culture experiment were harvested and concentrations of IL-10 and INF-γ were determined in duplicate using commercial ELISA kits following the manufacturer’s protocol (eBioscience).

### Protein extraction and Western blot

Purified MSCs (1 × 10^5^ cells/well) were lysed, and protein quantification was determined by a BCA kit (Thermo Fisher Scientific). Protein samples (4 μg/lane) were resolved by 10% SDS-PAGE, transferred to Immobilon®-P PVDF membrane (Millipore). The expression of IDO (Arigo, Cat#ARG59296), STAT3 (Cell signalling, Cat#9139) and p-STAT3 (Cell signalling, Cat#9131), GAPDH (GeneTex Inc., Cat#GTX100118), and β-actin (Sigma-Aldrich) were determined using specific antibodies. Bands were semi-quantified using ImageJ software (Version 1.52a), and GAPDH or β-actin acted as an internal reference.

### RNA preparation, semi-quantitative RT-PCR, and quantitative RT-PCR

Total RNA was extracted using the Trizol reagent (Invitrogen) and converted to cDNA using a ReverTra Ace set (Toyobo Life Science) according to the manufacturer’s instructions. PCR reaction was performed using the Ampliqon system (Ampliqon A/S). Bands were semi-quantified using ImageJ software (Version 1.52a), and GAPDH or β-actin acted as an internal reference. Real-time PCR analysis was performed using an ABI ViiA7 system (Applied Biosystems). For each sample, the cycle threshold (Ct) value was determined. The results were normalised to the levels of the GAPDH gene on the same plate. The level of mRNA expression for different cell groups was calculated using the 2ΔCt method. Primers specific for each gene were designed as follows: human β-actin, CTACGTCGCCCTGGACTTCGAGC (forward primer, F), GATGGAGCCGCCGATCCACACGG (reverse primer, R); human IDO, CGCTGTTGGAAATAGCTTC (F), CAGGACGTCAAAGCACTGAA (R). human GAPDH, GAGTCAACGGATTTGGTCGT (F), TTGATTTTGGAGGGATCTCG (R); human IL-10RA, CTCTGACAGTTGGCAGTGTGAAC (F), ACCTTGCGAATGGCAATCTC (R), IL-10, TCTCCGAGATGCCTTCAGCAGA (F), TCAGACAAGGCTTGGCAACCCA (R).

### Fluorescence-Activated Cell Sorting (FACS) analysis and cell sorting

MSCs (2 × 10^4^) and T cells (1 × 10^5^) were co-cultured with PHA (5 μg/mL) for 3 days. After co-culture, suspended cells were washed out. MSCs were then collected by trypsinization using trypsin-EDTA (GIBCO) and surface stained with anti-CD105-FITC (BD Biosciences, Cat#561443) and anti-IL-10R-PE (BD Biosciences, Cat#556013). The expression of human IL-10RA of MSCs was acquired using a Cytek Aurora flow cytometer (Cytek) and analysed by FlowJo software (10.8.0). For isolating IL-10RA positive MSCs, the CD105 and IL-10RA double-positive cells were sorted out using the BD Influx Cell Sorter system (BD Biosciences). The sorted MSCs were used in western blot analysis to determine the expression level of IDO.

### Preparation of IDO and IL-10RA knock-down MSCs

Lentiviral systems for gene silencing were obtained from the Taiwan National RNAi Core Facility. Sequences of short hairpin RNA (shRNA) in the pLKO.1 vectors are: pLKO.1-shLuc (5′-GCGGTTGCCAAGAGGTTCCAT-3′), pLKO.1- shIDO-a (5′-CAATCAGTAAAGAGTACCATA-3′), pLKO.1- shIDO-b (5′-CGTAAGGTCTTGCCAAGAAAT-3′), pLKO.1- shIL-10RA-a (5′-AGTGGCATTGACTTAGTTCAA-3′), and pLKO.1- shIL-10RA-b (5′-GTGAACCTAGAGATCCACAAT-3′). Lentiviruses were collected from media of 293FT cells co-transfected with pLKO.1-derived plasmids, packaging vectors pCMVdelR8.91 and pMD.G following the protocols provided by the Taiwan National RNAi Core Facility. MSCs (2 × 10^4^ cells) were pre-seed in 24-well plates for 16 h and then infected with lentiviruses at a multiplicity of infection (MOI) of 4 PFU per cell in the presence of protamine sulfate (8 μg/mL; Sigma). Cells were incubated for 24 h and replaced with puromycin (5 μg/mL) containing media for two days. Surviving MSCs were pooled for subsequent experiments. For T cell proliferation assay, MSCs (2 × 10^4^ cells) were co-cultured with allogeneic T cells (1 × 10^5^) in a 48-well plate. Additionally, MSCs (1 × 10^5^) were seeded in a 24-well plate and treated with allogeneic T cells (5 × 10^5^) for 3 days to determine RNA or protein levels of IDO.

### Preparation of Human PDAC organoids

Pancreatic ductal adenocarcinoma (PDAC) organoids were established using human primary PDAC tumour tissue obtained through surgical resection following the protocol [34]. Briefly, PDAC tumour tissue was mechanically dissociated into small tumour pieces and enzymatically digested using ACCUMAXTM (STEMCELL Technologies) (Sigma-Aldrich) and 10 μM Y-27632(Sigma-Aldrich), then embedded in 75% Matrigel Matrix (Corning) for standard used. The morphology of PDAC organoids and the effect of varying Matrigel concentrations on T cell infiltration in PDAC organoids are shown in Supplementary Fig. 4. Human PDAC organoids medium is composed of Advanced DMEM/F12 (GIBCO) supplemented with 10 mM HEPES (GIBCO), 1x glutaMAX (GIBCO), 1x Penicillin/Streptomycin (GIBCO), 100 ng/mL Noggin (Sigma), 10 ng/mL FGF-10 (Invitrogen), 100 ng/mL R-spondin 1 (R&D Systems), 1x B27 supplement without vitamin A (GIBCO), 1.25mM N-Acetylcysteine (Sigma-Aldrich), 10 mM nicotinamide (Sigma-Aldrich), 50 ng/mL human recombinant EGF (Peprotech), 50 ng/mL Wnt-3a (R&D Systems), 500 nM A83-01 (MedChemExpress), and 10 nM gastrin (MedChemExpress). Organoids were passaged approximately every two weeks by incubating in Cell recovery solution (Corning) and ACCUMAXTM (STEMCELL Technologies) for 10 min at 37°C to dissociate the Matrigel and organoids to single cells. The dissociated cells were then replated in fresh Matrigel matrix. After passaging, 10 μM Y-27632 was added to the medium for the first 2–3 days. Organoids were cryopreserved in 10% FBS/DMSO as master as well as working biobanks. Organoids between passages 2 to 10 were used in experiments. The utilisation of human PDAC samples in this research received approval from the Research Ethics Committee, National Health Research Institutes (EC1100308-E).

### Confocal Imaging of PDAC organoids

For live-image, PDAC organoids were seeded in 35 mm glass-bottom dishes (ibidi, Cat#81218-200) and cultured for 10 days in various concentrations of Matrigel (50%, 60%, 75% and 90%). CFSE-labelled human T cells were then added to the organoids, with or without the supplementation of PHA (5 μg/mL) and cultured for 5 days 37 °C, 5% CO_2_ incubator. Cells were then labelled with Hoechst 33342 (Sigma, B2261) and propidium iodide (PI, Sigma, Cat# P4170) before scanning with a Leica Stellaris 8 confocal spectral microscopy (Leica Microsystem, USA) using LEICA Application Suite X software (Version 4.3.0, 64-bit). Images were analysed with LAS X Office Software (Version 1.4.6 28433).

### Organoids-MSCs-lymphocytes co-culture

PDAC organoids were seeded in 24-well plates and grown to approximately 100 μm diameter size over 7 ~ 10 days in 75% Matrigel. The medium was replaced with an FBS-contained (5% v/v) PDAC organoid medium, and 10^4^ MSCs, IL-10RA-KD MSCs, and IDO-KD MSCs were seeded. After 4–5 h, 10^5^ CFSE-labelled human T cells were added with PHA (5 μg/mL). All cultured cells were maintained for 5 days in 37 °C, 5% CO2 incubator, the size of the PDAC organoids from various treated conditions was photographed and recorded, and the proliferating activity of co-cultured T cells in each group was assessed.

### Statistics

All data are presented as the mean ± standard error of the means (S.E.M.) from three independent experiments and analysed using an unpaired two-tailed Student’s *t*-test. All graphs were created, and statistical analysis was performed using GraphPad Prism (8.0.1). The results were considered statistically significant when *p* < 0.05.

## Results

### Correlation between up-regulation of kynurenine and IDO expressions and suppression of T cells proliferation in MSCs co-culture

**Fig. 1:**
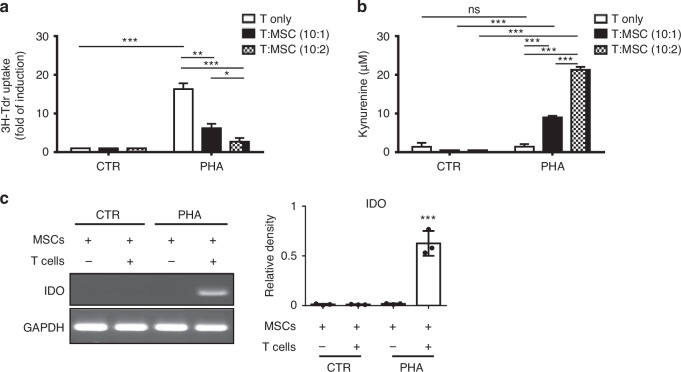
Suppression of T cell proliferation during human bone marrow-derived mesenchymal stem cells (MSCs) co-cultured accomplished increased kynurenine and Indoleamine 2,3-dioxygenase (IDO) expression. **a** T cells alone or co-cultured with MSCs at the indicated ratio were stimulated with phytohemagglutinin (PHA) (5 μg/mL) for 3 days. Proliferation was measured by [^3^H]-thymidine (1 μCi /well) incorporation after a 16-hour pulse. **b** The amounts of kynurenine in the culture supernatants were measured to determine the biological activity of IDO. **c** After co-culturing MSCs and T cells with PHA (5 μg/mL) at a ratio of 10:2 (T cells to MSCs), T cells were washed out and the RNA levels of IDO in MSCs were measured. Representative results of three independent experiments are shown in (**c**). Error bars, S.E.M. *n* = 3. (two-sided unpaired *t*-test, ns not significant, *p < 0.05, **p < 0.01, ***p < 0.001).

In the intricate interplay of the human immune system, the interaction between T cells and MSCs sparks a fascinating inquiry into their impact on T cell activity [30, 35]. Our study delves into this relationship by co-culturing MSCs with human CD3^+^ T cells and scrutinising the dynamic proliferation of T cells. We found that MSCs’ presence reduced cell proliferation upon phytohemagglutinin (PHA) activation in a dose-dependent manner, indicating MSCs’ nuanced modulation of T cell activity (Fig. 1a). Further analysis revealed increased kynurenine levels, indicative of IDO activity, in culture supernatants under PHA stimulation, correlating with MSCs ratio (Fig. 1b). Additionally, IDO gene expression in MSCs was upregulated only in T cells co-cultured with MSCs under PHA stimulation, confirming MSCs’ role in modulating T cell activity through IDO activity regulation (Fig. 1c).

### Up-regulation of IL-10, IL-10RA Expression and the Activity IDO in PHA-Activated T cells Contacted MSCs

**Fig. 2:**
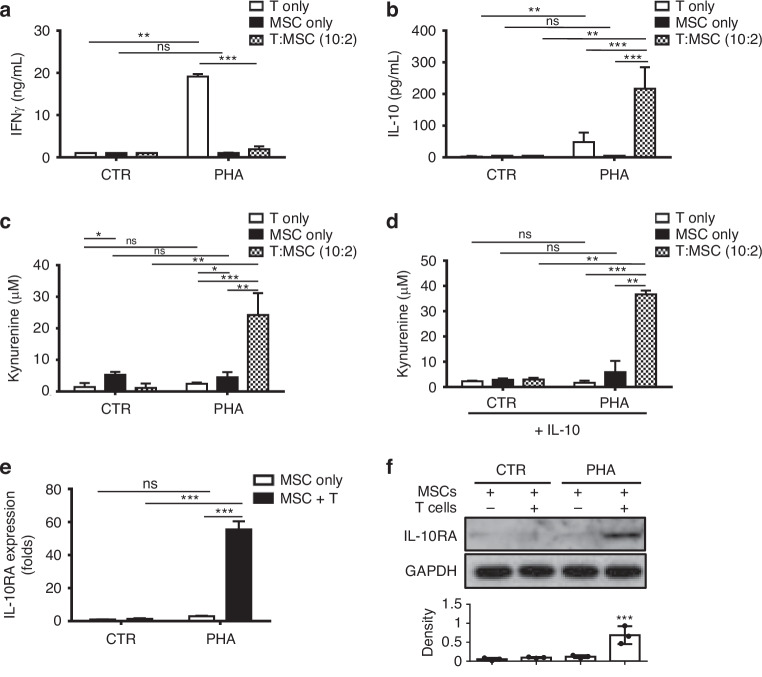
Increasing interleukin-10 (IL-10) production, IDO activity, and interleukin-10 receptor alpha (IL-10RA) expression during co-culture of MSCs and T cells. Production of IFN-γ (**a**), IL-10 (**b**) from MSCs and T cells co-culture (T cells to MSCs = 10:2) with the supplementation of PHA were measured by ELISA. (C&D) MSCs and T cells were cultured with PHA for 3 days in the absence (**c**) or presence (**d**) of additional IL-10 supplementation. Supernatants were collected to measure kynurenine levels. The MSCs and T cells were separated after culturing with PHA (5 μg/mL) (T cells to MSCs = 10:2), and the RNA (**e**) and protein (**f**) levels of IL-10RA were analysed in MSCs. The bar graph represents the densitometric analysis of IL-10RA expression normalised to GAPDH. Representative figure (top) and statistic (bottom) results of three independent experiments are shown. Error bars, S.E.M. *n* = 3. (two-sided unpaired t-test, ns, not significant, *p < 0.05, **p < 0.01, ***p < 0.00).

We observed significant T cell proliferation suppression with a 20% proportion of MSCs co-cultured. Subsequently, we analysed Interferon-γ (IFN-γ) levels, a T cell activation marker post-PHA stimulation (Fig. 2a). IFN-γ surging approximately 20-fold upon PHA stimulation in T cells alone. Intriguingly, the T:MSCs (10:2) set exhibited significant IFN-γ suppression post-PHA, suggesting MSCs’ potential influence on T cells’ IFN-γ production (Fig. 2a).

MSCs are prominent for their immunosuppressive and anti-inflammatory capabilities, primarily attributed to IL-10 or IDO secretion [16, 21]. Analysing IL-10 concentration from MSCs with or without T cells showed a significant disparity between PHA-activated T cells alone (T only) and the control T cell-only set (CTR) (Fig. 2b). Both MSCs-only groups, whether stimulated with PHA or not, exhibited similar basal levels of IL-10 expression (Fig. 2b). Consequently, co-culture MSCs with T cells post-PHA stimulation led to a more than threefold increase in IL-10 concentration (Fig. 2b), highlighting MSCs’ capacity to suppress T cell activation through IL-10 secretion, consistent with prior research [16, 17].

Additionally, baseline levels of kynurenine production, indicating IDO expression, were observed in the T only, MSC only, and T:MSCs (10:2) groups under separate PHA treatments (Fig. 2c). However, in the T:MSCs (10:2) group exhibiting reduced IFN-γ secretion, kynurenine levels increased approximately 25-fold post PHA stimulation, indicating the involvement of IFN-independent mechanisms. Consequently, we redirected our attention to the elevated IL-10 levels in the environment when MSCs suppressed T cells. To verify IL-10’s role in regulating kynurenine production, additional IL-10 was introduced to the same three groups of cells under PHA-free or PHA-stimulated conditions. Our results reveal that IL-10 alone failed to promote kynurenine production in T cells or MSCs alone, emphasising its insufficient action to induce IDO expression and the necessity of T:MSCs co-culture under PHA for kynurenine induction (Fig. 2d).

To decipher the key factors triggering IDO expression via IL-10 signalling, we observed that up-regulation of IL-10 receptor A (IL-10RA) in T:MSCs (10:2) culture at both RNA and protein levels following PHA activation (Fig. 2e, f). Quantitative PCR revealed a 60-fold increase in IL-10RA expression exclusively in T:MSCs (10:2) cultures (Fig. 2e). This elevation was further corroborated at the protein level, where IL-10RA expression was only detected in the T:MSCs (10:2) culture set, with GAPDH as the loading control (Fig. 2f). We further examined the expression level of IL-10 and IL-10 RA in T cells and MSCs separately after co-culturing. The qRT-PCR results revealed that MSCs predominantly express IL-10 and IL-10RA when co-cultured with T cells in the presence of PHA (Supplementary Fig. 2b–e). Collectively, this heightened expression of IL-10RA highlights its pivotal role in T cell suppression within T:MSC co-culture environment.

### IDO induction in IL-10RA^+^ MSCs in MSCs/T cells coculture

**Fig. 3:**
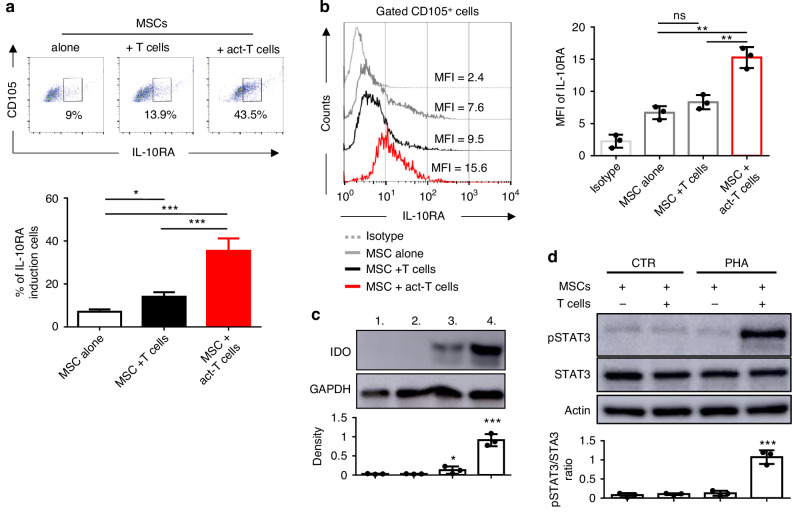
IL-10RA^+^ MSCs from the T cells co-cultures exhibit a significant induction of IDO protein. **a**, **b** Induction of IL-10RA in T cells contacted MSCs were measured by flow cytometry. **a** The percentage of IL-10RA-induced CD105^+^ population (CD105^+^IL-10RA induction cells) is shown. Representative flow cytometry data are presented in the top panel, while cumulative data from three independent experiments are displayed in the bottom panel. **b** The intensity of IL-10RA in CD105^+^ MSCs is shown. Represent flow data are shown in the left panel, with cumulative data from three independent experiments presented in the right panel. The isotype control is included for comparison. **c** Protein levels of IDO were measured by western blot in control MSCs (Lane 1), sorted CD105^+^ cells after co-culture MSC with non-activated T cells (Lane 2), and sorted CD105^+^ cells (Lane 3) or CD105^+^IL-10RA induction cells (Lane 4) after co-culture MSCs with activated T cell. The ratio for co-culture MSCs with T cells was 2:10 with the supplementation of PHA and IL-10. The bar graph represents the densitometric analysis of IDO expression normalised to GAPDH. Representative (top) and statistical (bottom) results of three independent experiments are shown. **d** The expression of STAT3 and phosphorylated STAT3 (pSTAT3) were assayed in MSCs after co-culture with T cells via western blot. MSCs were co-cultured with T cells at the ratio of 2:10 with or without the presence of PHA for 3 days. The pSTAT3/STAT ratio from three independent experiments is shown in representative (top) and statistical (bottom) results. Error bars, S.E.M. *n* = 3. (two-sided unpaired *t*-test, ns not significant, *p < 0.05, **p < 0.01, ***p < 0.001).

To elucidate IL-10RA expression in MSCs within T: MSCs cocultured, CD105 and IL-10RA were utilised as MSCs’ markers to distinguish MSCs across three cell cultures. FACS analysis showed that approximately 9% of CD105^+^MSCs expressed IL-10RA alone, increasing to 13.9% with T cells co-cultured and surging to 43.5% with PHA-activated T cells (Fig. 3a, upper panel). The average percentage of IL-10RA induction MSCs increased from 7.1% to 14.1% with T cells co-cultured and further to 35.5% with PHA-activated T cells (Fig. 3a, lower panel).

Sorting CD105^+^ MSCs and assessing IL-10RA expression by mean fluorescence intensity (MFI) revealed an MFI of 7.6 when MSCs were alone, which slightly increased to 9.5 without statistical significance in co-culture with T cell but rose significantly to 15.6 in the presence of PHA-activated T cells (Fig. 3b, left). Quantitative MFI data showed significant increases in IL-10RA expression in CD105^+^ MSCs when cocultured with PHA-activated T cells compared to MSCs alone or cocultures with non-activated T cells (Fig. 3b, right). These results suggest that IL-10RA was primarily upregulated in MSCs when co-cultured with PHA-activated T cells, particularly in the presence of IL-10 supplementation in the co-culture environments.

Western blot analysis confirmed non-detectable IDO expression in MSCs alone, (Fig. 3c, lanes 1 and 2), detectable expression in CD105^+^ MSCs (Fig. 3c, lane 3), and higher expression in IL-10RA^+^ MSCs (Fig. 3c, lane 4). Given that IL-10RA was only expressed in a subset of CD105^+^ MSCs in T:MSCs co-culture (Fig. 3a, b), the high IDO expression in CD105^+^IL-10RA^+^ MSCs suggests an IL-10RA-dependent regulation of IDO in MSCs (Fig. 3c). Previous studies have highlighted the crucial role of STAT3 signalling in regulating IL-10 signalling or IDO expression [36]. Phosphorylated STAT3 (pSTAT3) was detected exclusively when MSCs were co-cultured with PHA-activated T cells (Fig. 3d), suggesting that activated STAT3, particularly pSTAT3, functions exclusively in MSCs and may potentially regulate IDO expression.

### Depletion of IL-10RA in MSCs Diminishes IDO Activity in Co-Culture with Activated T Cells

**Fig. 4:**
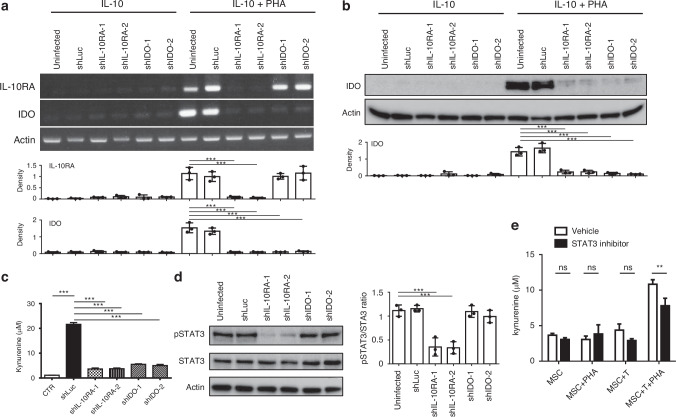
Inhibition of IL-10RA expression in MSCs decreases IDO activity during co-culture with activated T cells. MSCs were infected with shLuc (as a non-targeting control), shIDO-1, shIDO-2, shIL-10RA-1, and shIL-10RA-2 lentiviruses and selected for infected cells in the puromycin (5 μg/mL) containing medium for 2 days. The infected MSCs were then co-cultured with allogeneic T cells at the ratio of 2:10 in the presence of PHA and IL-10. MSCs and T cells were separated after culture. **a** RNA levels of IL-10RA and IDO were measured via RT-PCR. The bar graphs represent densitometric analyses of IL-10RA and IDO expression normalised to Actin. Representative image (top) and statistical analysis (bottom) from three independent experiments are shown. **b** Protein levels of IDO in MSCs were measured by western blot. The bar graph represents the densitometric analysis of IDO expression normalised to Actin. Representative image (top) and statistical analysis (bottom) from three independent experiments are shown. **c** The bioactivity of IDO was determined by measuring the amount of kynurenine from the cultured supernatant. **d** MSCs infected with shLuc, shIDO-1, shIDO-2, shIL-10RA-1, and shIL-10RA-2 lentiviruses were cultured with T cells at the ratio of 2:10 in the presence of PHA and IL-10. The MSCs and T cells were separated after culturing, and the expression of STAT3 and phosphorylated STAT3 (pSTAT3) in MSCs are shown. The pSTAT3/STAT ratio from three independent experiments is shown in representative (left) and statistical (right) results. **e** The combination of MSCs co-cultured with T cells and PHA treatment was carried out as indicated. On the second day of co-culture, T cells were washed out, the medium was refreshed, and 1 ng/mL of IL-10 and 0.1 μM of STAT3 inhibitor 4 m or vehicle were added to the MSCs, which were then cultured for one additional day. The medium was harvested and used for a kynurenine assay to assess IDO activity. Error bars, S.E.M. *n* = 3. (two-sided unpaired *t*-test, ns not significant, *p < 0.05, **p < 0.01, ***p < 0.001).

The upregulation of IL-10RA in MSCs, associated with T cells inactivation, underscores the pivotal role of IL-10RA-mediated immunosuppression. To explore the significance of IL-10/IL-10RA signalling in MSC-mediated T cell inactivation, lentiviral-mediated shRNA was utilised to target IL-10RA and IDO genes in MSCs, leading to their silencing. Following co-culture with allogeneic T cells and additional IL-10 with or without PHA stimuli, IL-10RA and IDO expressions were evaluated via semi-quantitative PCR. IL-10RA knockdown significantly reduced both IL-10RA and IDO expressions in MSCs, particularly under PHA activation with additional IL-10 (Fig. 4a), suggesting a correlation between IL-10RA and IDO expression. Conversely, IDO gene knockdown did not influence IL-10RA expression, indicating that IDO expression is downstream of IL-10RA (Fig. 4a). Subsequent protein-level analysis validated this correlation (Fig. 4b), while quantification of kynurenine concentration in co-cultured supernatants revealed kynurenine secretion only in PHA-activated T cells co-cultured with shLuc-MSCs, indicating a significant reduction compared to controls (Fig. 4c). These findings underscore the roles of both IL-10RA and IDO in MSCs in upregulating kynurenine secretion, with IL-10RA acting as an epistatic factor of IDO expression, highlighting the intricate interplay between IL-10/IL-10RA signalling and IDO-mediated immunosuppression in MSCs-mediated T cells inactivation.

Moreover, when IL-10RA or IDO genes were silenced by shRNA, along with uninfected and shLuc controls, and co-cultured with PHA-activated T cells, the levels of pSTAT3 were reduced in both shIL-10RA MSCs (Fig. 4d). To understand the importance of STAT3 activity in regulating IDO gene expression through IL-10RA, we introduced a STAT3 inhibitor into the co-culture system. The cytotoxicity of the STAT3 inhibitor on MSCs was evaluated to determine a non-lethal concentration (Supplementary Fig. 3). Given the critical role of STAT3 in T cell function, T cells were removed after co-culture, and a STAT3 inhibitor was applied to the MSCs. IDO activity was subsequently measured using a kynurenine assay. The results indicated that IDO activity in MSCs was inhibited following STAT3 inhibitor treatment in the presence of T cells (Fig. 4e). These findings confirm that IL-10RA regulates IDO gene expression by acting upstream of STAT3.

### Rejuvenation of T cell activation and proliferation proficiency through IL-10RA downregulation in MSCs during co-cultivation

**Fig. 5:**
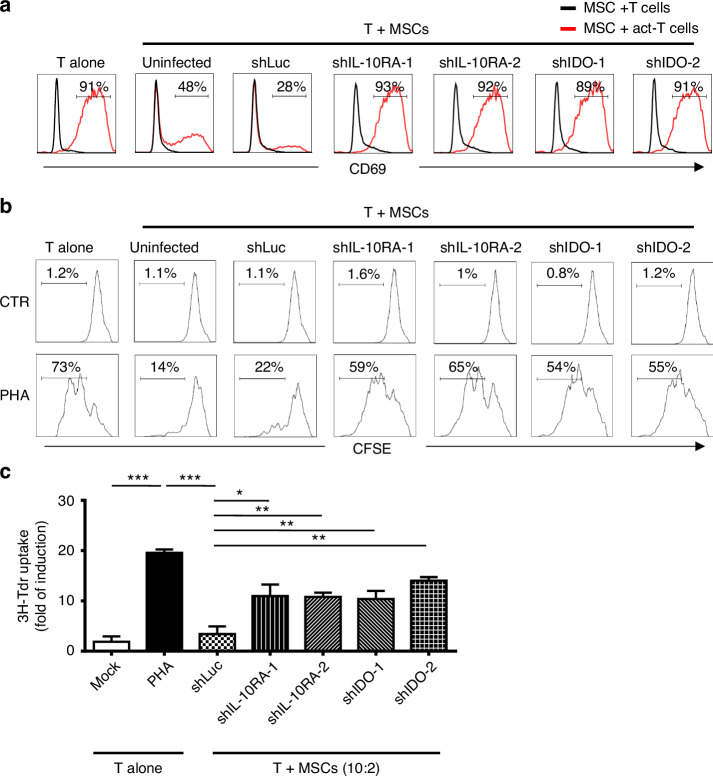
Knockdown of IL-10RA expression in MSCs restores T cell activation and proliferation during co-culture. **a** The activation marker CD69 on T cells was analysed by flow cytometry after co-culturing with shLuc, shIDO, or shIL-10RA-MSC at a 2:10 ratio, supplemented with PHA and IL-10 for 3 days. Carboxyfluorescein succinimidyl ester (CFSE) proliferation (**b**) and ^3^H-thymidine incorporation assay (**c**) were used to monitor T cell proliferative activity after co-culture with indicated MSCs. Error bars, S.E.M. *n* = 3. (two-sided unpaired *t*-test, ns not significant, *p < 0.05, **p < 0.01, ***p < 0.001).

To further validate the impact of IL-10RA and IDO on T cell activation in T:MSCs co-culture, four IL-10RA and IDO knockdown clones expressed in MSCs (shIL-10RA-1, shIL-10RA-2, shIDO-1, and shIDO-2), along with shLuc and uninfected negative controls, were co-cultured with PHA-activated or non-PHA-activated T cells, supplemented with IL-10. CD69 expression in CD3^+^ T cells revealing that knocking down IL-10RA or IDO expressions in MSCs restored T cell activation to around 90% compared to the activated-T alone or uninfected/shLuc controls (Fig. 5a).

Moreover, we explored the influence of IL-10RA on IDO and its role in MSCs’ ability to inhibit T cell activation. T cell proliferation dropped from 73% to 14–22% when co-cultured with uninfected or shLuc MSCs but rebounded to 54–65% upon knocking down IL-10RA or IDO with PHA-activated T cells. (Fig. 5b, lower panel). This underscores the significance of both IL-10RA and IDO in MSCs for suppressing T cell proliferation. Thymidine incorporation assays confirmed restored proliferation in T cells when IL-10RA and IDO were knocked down in surrounding MSCs (Fig. 5c). Thus, from CD69 expression to CFSE proliferation and ^3^H-thymidine incorporation assays, these findings underscore the importance of IL-10RA and IDO in suppressing T cell activation in MSCs, emphasising the regulatory role of IL-10RA on IDO expression and its consequent impact on T cell activity.

### Revitalisation of T cell lethal efficacy against PDAC organoids via IL-10RA downregulation in MSCs during co-cultivation

**Fig. 6:**
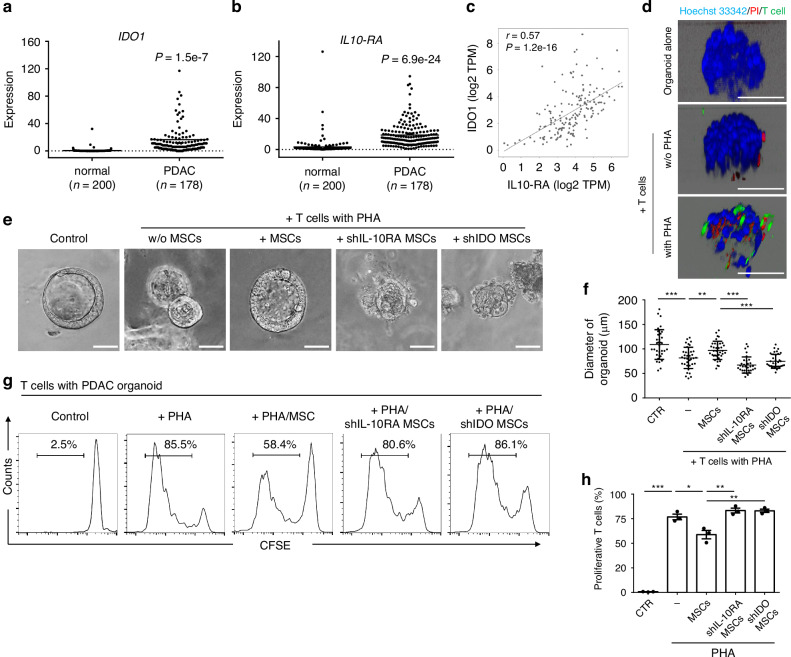
Knockdown of IL-10RA expression in MSCs restores T cells’ killing activity on PDAC organoids upon co-culture. Expression of *IDO1* (**a**) and *IL-10RA* (**b**) in normal human pancreas and pancreatic ductal adenocarcinoma (PDAC) tissue samples. **c** Correlation analysis between IDO1 and IL-10RA gene expression levels. **d** Human PDAC organoids were co-cultured with CFSE-labelled human T cells in the presence or absence of PHA supplementation (5 μg/mL) for 5 days. Hoechst 33342 (blue) and propidium iodide (PI, red) were used to stain total cells and dead cells (PI-positive cells), respectively, following co-culture. Confocal microscopy was employed to directly scan the labelled cells, enabling visualisation of the organoids and T cells in contact with the organoids (green). Scale bar, 100 μm. **e**, **f** Human PDAC organoids were co-cultured with human T cells in the presence of parental MSCs, IL-10RA knockdown MSCs, IDO knockdown MSCs, and PHA (5 μg/mL) for 5 days, maintaining an MSC-to-T cell ratio of 1:10. Panel (**e**) displays representative images of PDAC organoid morphology post co-culture, while panel (**f**) presents statistical analysis of organoid diameters (μm). Error bars indicate S.E.M. from three independent experiments (*n* = 3). Statistical significance was determined using a two-sided unpaired *t*-test (**p < 0.01, ***p < 0.001). Scale bar, 100 μm. **g**, **h** To assess T cell proliferative activity after co-culture, human T cells were pre-labelled with CFSE and analysed via flow cytometry. Panel (**g**) shows representative results of the percentage of proliferating T cells relative to total T cells, and panel (**h**) provides the corresponding statistical analysis. Error bars, S.E.M. *n* = 3. (two-sided unpaired *t*-test, *p < 0.05, **p < 0.01, ***p < 0.001).

The battle against cancer relies on unravelling the intricate dynamics between the immune response and the tumour microenvironment (TME). To comprehend the connection between IL-10RA and IDO expression levels within the TME, we analysed clinically relevant data from The Cancer Genome Atlas (TCGA) via the OncoDB website (https://oncodb.org/). Our results validate improved IL-10RA and IDO expression in human pancreatic ductal adenocarcinoma (PDAC) tumours (Fig. 6a,b), and reveal their robust correlation (Fig. 6c). A central aspect of our study involved elucidating how IL-10 receptor activation influences IDO expression, thereby impacting T cell cytotoxicity against tumours. To achieve this, we meticulously co-cultured PDAC organoids with stromal cells (MSCs) subjected to various modifications, revealing the nuanced effects of IL-10RA and IDO on T cell functionality within the realm of immuno-oncology (Fig. 6d–h and Supplementary Fig. 4). We established PDAC organoids using varying Matrigel concentrations (50%, 60%, 75%, 90%) and then co-cultured with T cells, either with or without PHA activation, to assess T cells-mediated tumour cytotoxicity. Organoids formed well-defined spheroid structures only at Matrigel concentrations greater than 50%. When co-cultured with non-PHA-activated T cells, only a few PI-positive dead cells appeared at the edges of organoids with 60% and 75% Matrigel. Notably, regardless of Matrigel concentration, organoids co-cultured with PHA-activated T cells consistently resulted in a pronounced signal of PI-stained dead cells (in red) and significant infiltration of CFSE-labelled T cells (in green), with infiltration decreasing as Matrigel concentration increased. Our findings confirm that activated T cells possess a heightened ability to infiltrate tumours and induce cell death within the tumour microenvironment. Particularly, fewer T cells were found in 90% Matrigel organoids, suggesting that higher Matrigel concentrations may impede T cell infiltration despite PHA activation. Given the higher stiffness of human PDAC samples, we selected 75% Matrigel for PDAC organoid formation and T cell infiltration assay (Fig. 6d and Supplementary Fig. 4). We then examined PDAC organoids co-cultured with PHA-activated T cells alone or with MSCs, IL-10RA knockdown MSCs (shIL-10RA), and IDO knockdown MSCs (shIDO). Notably, PDAC organoid size significantly diminished when exposed to T cells in the absence of MSCs or when co-cultured with IL-10RA or IDO silencing MSCs, but enlarged when in contact with immuno-suppressive control MSCs (Fig. 6e), underscoring the crucial role of IL-10 signalling pathway in orchestrating TME-mediated immune evasion. Quantitative analysis of PDAC organoid diameter corroborated these observations (Fig. 6f), showing reduced size when surrounded by T cells or PHA-activated T cells and when IL-10RA or IDO expressions were suppressed in surrounding MSCs.

Further exploration into T cell proliferation dynamics shed light on MSCs-mediated immunosuppression, with PHA activation sparked robust T cell proliferation (from 0.9% to 82.3%), which was dampened by co-culture with MSCs (declining to 50.7%) (Fig. 6g) Notably, ablation of IL-10RA and IDO expression in MSCs restored T cells proliferation to near-normal levels (86.1% ~ 87.9%), signifying a significant reversal of immune suppression within the TME (Fig. 6g). Statistically analysed, MSCs expressively inhibited T cell proliferation, the suppression that was restored upon IL-10RA and IDO knocking down (Fig. 6h). This highlights the crucial role of the engagement of IL-10/IL-10RA signalling and the expression of IDO in orchestrating TME-mediated immune evasion, correlating with T cell activation status. Overall, this emphasised the determinative roles of IL-10RA and IDO in MSCs to modulate T cell function within the TME. Taken together, our findings propose a promising approach for therapeutic intervention, suggesting that targeting IL-10RA or IDO expression in tumours-surround stromal cells may unleash the full potential of T cells-mediated tumour eradication, renewing hope in precision immunotherapy against cancer.

## Discussion

IDO assumes a crucial role in orchestrating T cell activation and proliferation by modulating the kynurenine pathway of tryptophan metabolism, yielding immunosuppressive tryptophan metabolites such as kynurenine and 3-hydroxy anthranilic acid. [37, 38]. Tumour cells expressing IDO modulate immune responses, leading to reduced T cell infiltration and enhanced accumulation of regulatory T cells [6], which correlates with poor prognosis across different cancers [9]. Several small-molecule candidates and peptide vaccines have demonstrated significant tumour regression, with ongoing clinical trials targeting IDO activity or its expression [39]. Epacadostat, a highly specific inhibitor targeting IDO1, showed promise in phase I/II trials when combined with anti-PD1 therapy [10, 40]. However, in the phase III trial, combining epacadostat with pembrolizumab for advanced melanoma patients did not show superiority over pembrolizumab alone [41]. Subsequently, the trial was terminated, marking a serious setback in the clinical development of IDO1 inhibitors, with several phase III trials halted or downgraded to phase II [39]. Therefore, IDO remains a viable therapeutic target to counteract immune suppression in the TME for certain trials targeting specific cancer types such as head and neck, glioblastoma, and bladder cancers [42]. The quest for more effective IDO inhibitors or regulators remains imperative. In our study, we revealed the intricate involvement of the IL-10RA-mediated signalling pathway in IDO expression and its bioactivity, elucidating the immunoregulatory network between IDO and IL-10RA pathways. This suggests that IL-10RA could serve as an alternative target for efficiently modulating immunity through the regulation of IL-10 signalling and IDO expression, particularly relieving the immune-suppressive interaction of tumour cells/stromal cells and cytotoxic T cells with TME. The expression level of IL-10RA in the TME, along with its impact on IDO expression and activity, may be more suitable as biomarkers for cancer treatment and prognosis, representing a more promising future.

IFN-γ serves as a crucial inducer for IDO expression and exhibits potential in MSCs system, which has been shown to modulate immunity through IDO expression. However, concerns arise regarding the adequacy of IFN-γ alone to achieve optimal immunosuppressive ability within the MSCs or stromal cells involved in immunomodulatory microenvironment. This necessitates a deeper exploration of other mechanisms regulating IDO in this context. Previous studies have highlighted the effectiveness of combining inflammatory cytokines, such as TNF-α, IL-1α or IL-1β, with IFN-γ to induce the immunosuppressive capability of MSCs effectively [43]. This suggests that while IFN-γ plays a critical role, it may not be sufficient for inducing IDO expression in an immunosuppressive microenvironment. Evidence also indicates that existence of an IFN-γ-independent pathway for IDO expression. In a subset of murine DCs with IFN-γ receptor knockout, T cell suppression was inhibited by blocking the CTLA-4 mediated pathway, and supplementation of IDO inhibitor, 1 methyl-D-tryptophan (1-MT), reversed the suppressive effect [44], which represents an IDO-dependent T cell inhibition through an IFN-γ-independent pathway. The traditional notion that elevated IFN-γ expression triggers IDO expression might lead to clinical trials incorporating IFN-γ as part of enrollment criteria, neglecting the role of IL-10/IL-10RA. The correlation between *IDO1 and CXCL9* (as an IFN-γ responsive marker) has been assessed in colorectal carcinomas, melanomas, and other cancer types (10), but the correlation between *IDO* and *IL-10RA* in real tumours remains poorly elucidated. Our study reveals a mechanism involving an anti-inflammatory factor in inducing IDO expression and function throughout by IL-10/IL-10RA modulation. This oversight could impact the future development of IDO inhibitors or prompt a shift towards targeting IL-10RA as a novel therapeutic approach.

Modulating the expression and function of IDO could offer a novel therapeutic avenue, not only for cancer treatment but also for addressing IBD, a T-cell-mediated pathogenesis, which currently lacks effective drug therapies. Reports indicate that the induction of IDO limits the severity of experimental IBD [45], while inhibition of IDO augments T helper 1 cell (Th1)-mediated trinitrobenzene sulfonic acid (TNBS) colitis [46] underscoring the regulatory impact of IDO on gastrointestinal immune responses. The polymorphisms of IL-10RA have been associated with the pathogenesis of IBD, implying the involvement of the IL-10/IL-10RA pathway in IBD [47]. Our data establishes a link from IL-10RA regulation to IDO expression and function, suggesting that IL-10RA polymorphisms may not only influence the IL-10/IL-10 receptor pathway but also control IDO activity in the development of early-onset IBD. It succinctly emphasises the importance of understanding the relationship between IL-10RA expression and IDO activity in various contexts, such as inflammatory diseases, infection control, and cancer immunotherapy.

The immunosuppressive effects of MSCs have been extensively studied in both GvHD and organ transplantation, showcasing their broad capacity for immune regulation (14). In this study, due to research limitations, we analysed tumour cytotoxicity using allogeneic T cells against PDAC. Despite differences from the autologous immune response, it was evident that MSCs in the tumour environment effectively suppressed T cell activity. Targeting IL-10 and IDO was sufficient to counteract the significant impact of stromal cells on TME immune suppression. Our research introduces a novel immunotherapeutic approach for PDAC treatment- blocking IL-10R signalling to decrease IDO expression.

PDAC remains highly lethal, with a mere 5% 5-year survival rate, largely resistant to standard therapies [48]. Its grim prognosis is attributed to an extensively fibrotic and immunosuppressive TME, characterised by stromal cells, regulatory T cells, M2 macrophages, and fibroblasts that inhibit anti-tumour immunity [49]. Recent efforts have focused on combination immunotherapies aimed at reversing stromal cell-mediated immunosuppression and enhancing T cell cytotoxicity. These strategies include checkpoint inhibitors, reprogramming pro-tumour macrophages, and inhibiting TME-derived factors such as IDO, TGF-β, and CXCL12 [50]. By demonstrating that IL-10R signalling blockade reduces IDO expression and consequently alleviates immunosuppression from IL10/IDO axis-related stromal cells, our findings offer promising therapeutic avenues for disarming PDAC’s immunosuppressive TME, presenting an immunomodulatory axis for intervention.

## Supplementary information

Supplementary data

## Data Availability

Data are available on reasonable request.
