# Supplementary material for: IL-10RA governor the expression of IDO in the instruction of lymphocyte immunity
Source: Br J Cancer. 2024 Nov 26;132(1):126–36. doi: 10.1038/s41416-024-02893-3 (PMC11723913; doi:10.1038/s41416-024-02893-3)
Supplement: Supplementary file 1 — Supplementary data [file 41416_2024_2893_MOESM1_ESM.docx]

**Supplementary data**

**IL-10RA governor the expression of IDO in the instruction of lymphocyte immunity**

Tzong-Shyuan Tai, Duen-Wei Hsu, Yu-Shao Yang, Jai-Wen Shi, Ching-Yen Tsai, Chien-Hui Wu, Shu-Ching Hsu

**Supplementary Figure 1: Characterization of human bone marrow-derived MSCs.** **(A)** Morphology of cultured MSCs. **(B)** Expression of human CD34-, CD45-, CD11b-, CD90-, CD73-, CD105-, and CD49b markers were validated. MSCs were uniformly negative for human CD34-, CD45-, and CD11b, and positive for human CD90-, CD73-, CD105-, and CD49b. Scale bar, 50 μm


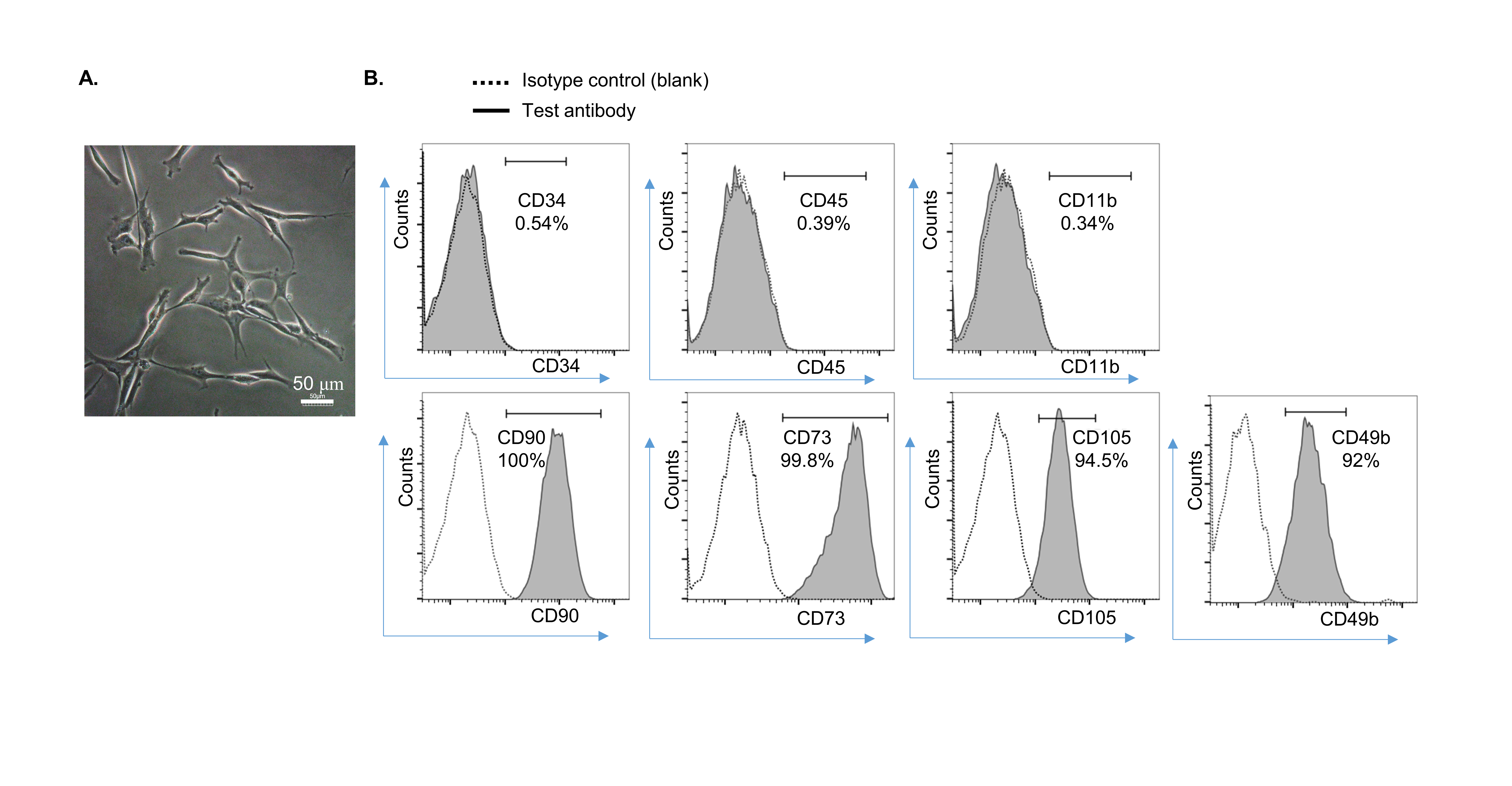


**
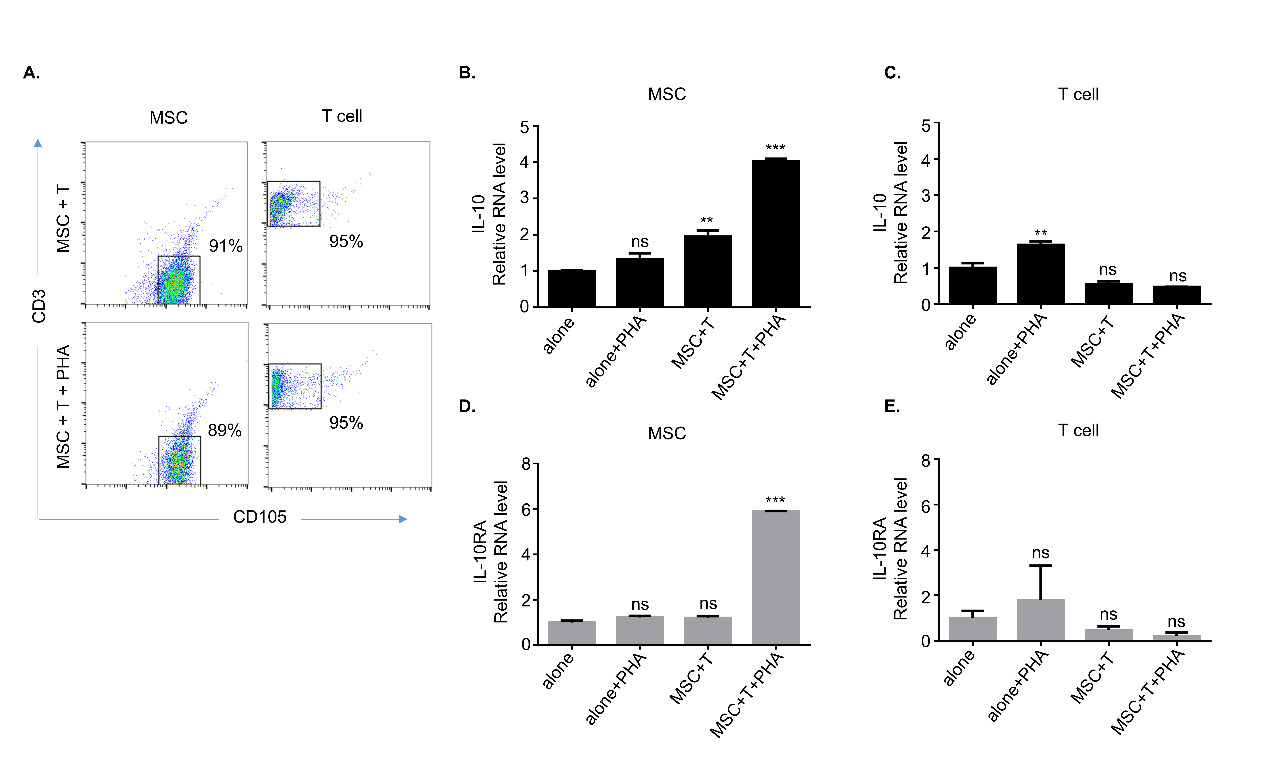
Supplementary Figure 2:** **MSCs predominantly express IL-10 and IL-10 RA when co-cultured with T cells under PHA activation.** **(A)** MSCs were co-cultured with allogeneic T cells at a 2:10 ratio for 3 days, with or without PHA in the presence of IL-10. After co-culture, MSCs and T cells were separated, and cell purity was confirmed by FACS analysis. The expression level of IL-10 and IL-10RA in the treated MSCs and T cells were assessed using qRT-PCR **(B-E)**. Error bars, S.E.M. n=3. (two-sided unpaired t-test, ns, not significant, **p < 0.01, ***p < 0.001)


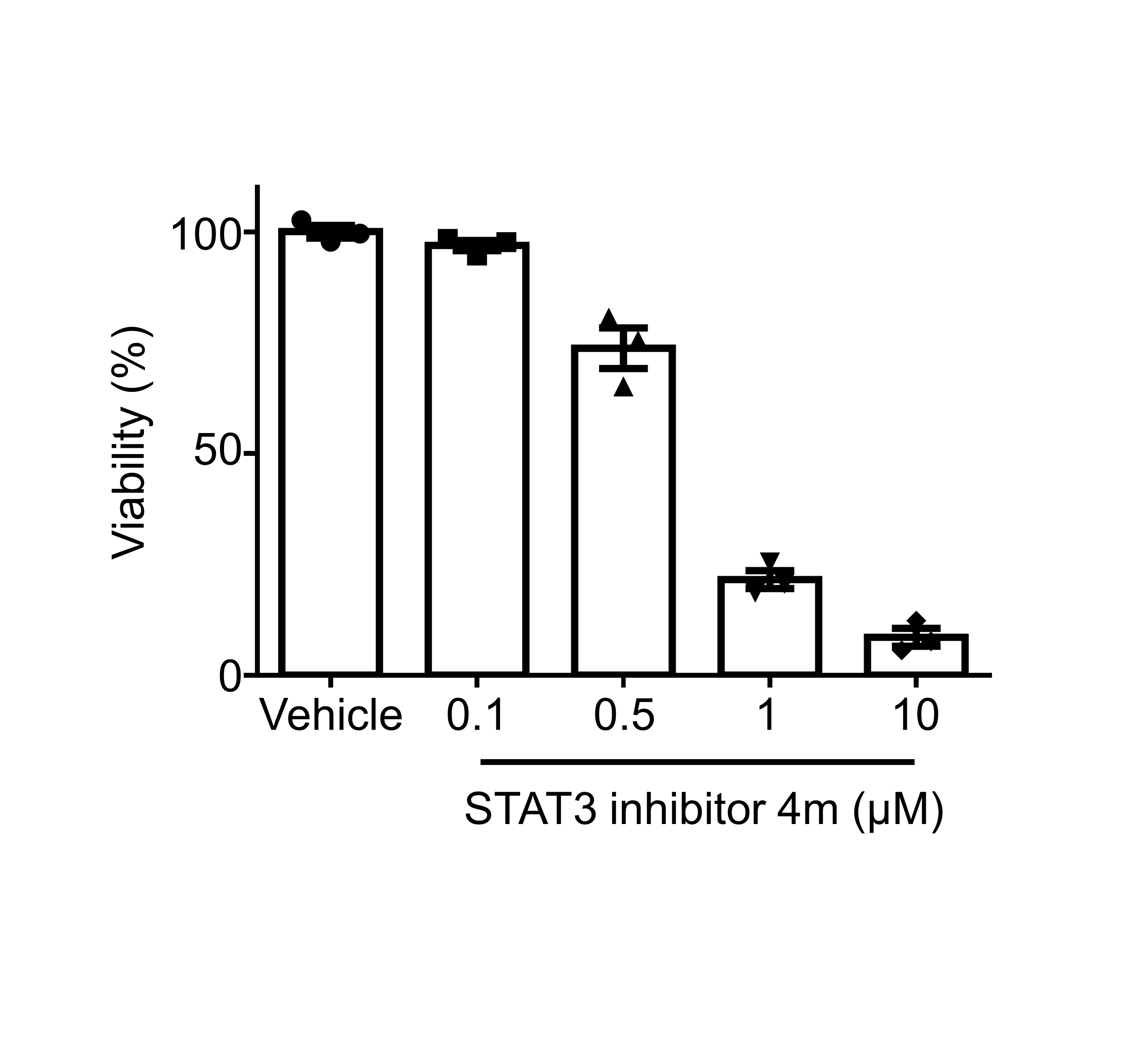


**Supplementary Figure 3: Viability assessment of MSCs Under STAT3 Inhibitor 4m Treatment.** MSCs were exposed to a range of STAT3 inhibitor 4m (Cayman, Cat#37352) concentrations (0.1, 0.5, 1, and 10 μM) over a three-day period. Cell viability was quantified using the highly sensitive WST-1 assay kit (Roche, Cat#5015944001), with survival percentages calculated relative to the vehicle-treated group. The results demonstrate a dose-dependent effect of STAT3 inhibitor 4m on MSC viability. Notably, at the lowest concentration tested (0.1 μM), we observed no significant reduction in MSCs viability compared to the control, which will be applied in further experiments. Error bars represent S.E.M. (n=3).

(A)


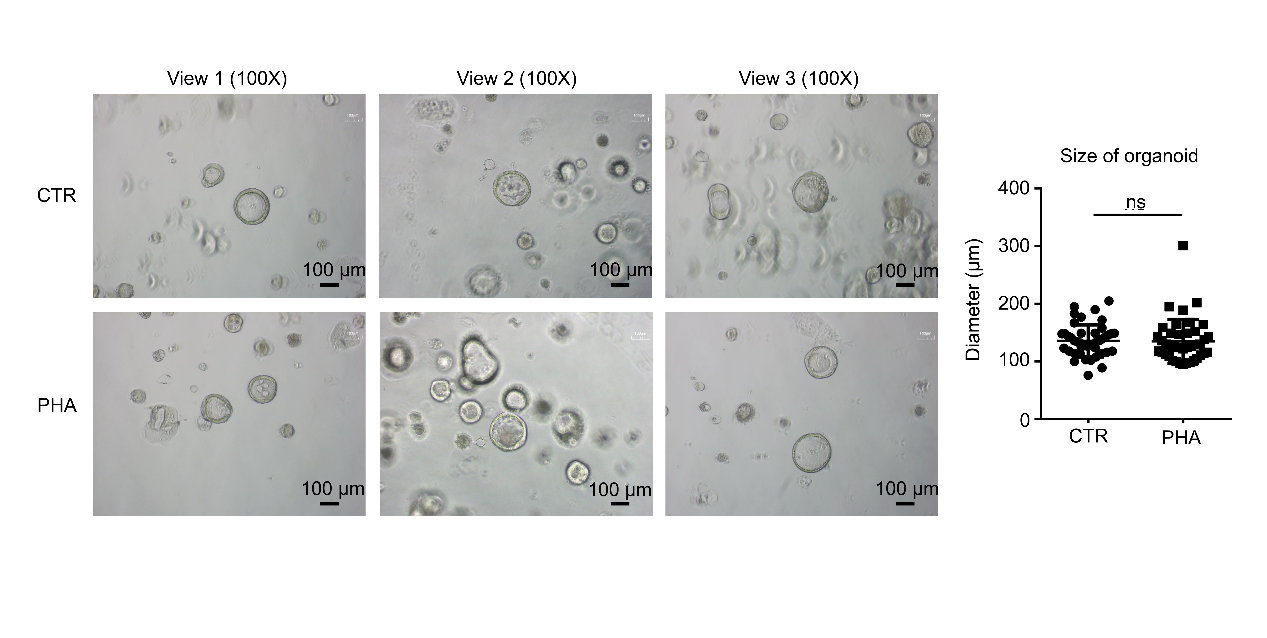


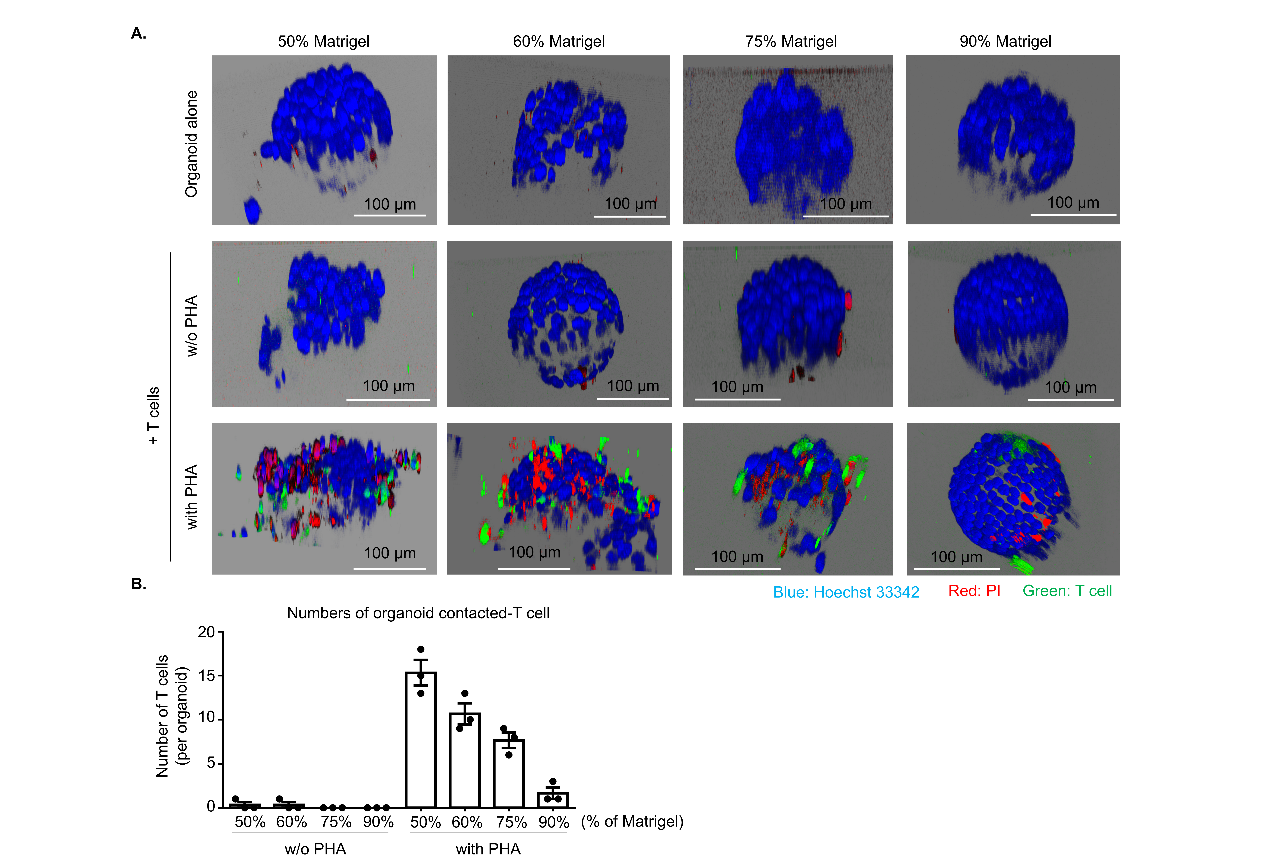
(B)

**Supplementary Figure 4:** **Interaction Dynamics between T Cells and PDAC Organoids under Varying Conditions (A)** Morphology of PDAC organoids with or without PHA (5 μg/mL) for three days. Quantitative analysis revealed no statistically significant differences in organoid formation, expansion, or morphogenesis between control and PHA-treated groups. Error bars, S.E.M. n=3, (two-sided unpaired t-test, ns, not significant). **(B)** T cell infiltration and organoids cell death assessment. CFSE-labeled human T cells (green) were added to the PDAC organoids with or without 5 μg/mL PHA for 3 days. When non-activated T cells were added to organoids formed with different concentrations of Matrigel, a small amount of PI-stained dead cells (in red) appeared at the edges of organoids treated with 60% and 75% Matrigel. However, co-culturing organoids with PHA-activated T cells resulted in significant T cell infiltration into the organoids, with the infiltration rate decreasing as Matrigel concentration increased. High levels of cell death signals were observed within organoids treated with PHA-activated T cells. In 90% Matrigel, fewer T cells were found within the organoids, suggesting that higher Matrigel concentrations may impede T cell infiltration even with PHA activation. In summary, under standard conditions (75% Matrigel), T cells can successfully infiltrate organoids and induce cell death. Matrigel concentration significantly affects T cell infiltration, with a marked decrease observed at concentrations above 90%. The number of T cells in contact with organoids was also quantified, showing a negative correlation between T cell numbers and Matrigel concentration (n=3). Scale bars are shown in the graph.
